# Health-related social control in overweight romantic couples: daily associations with physical activity and affect for targets and agents

**DOI:** 10.1093/abm/kaae093

**Published:** 2025-01-08

**Authors:** Pascal Küng, Corina Berli, Patrick S Höhener, Robert Tobias, Urte Scholz

**Affiliations:** Department of Psychology, University of Zurich, CH-8050 Zurich, Switzerland; Institute of Psychology, University of Bern, CH-3012 Bern, Switzerland; Department of Psychology, University of Zurich, CH-8050 Zurich, Switzerland; Department of Psychology, University of Zurich, CH-8050 Zurich, Switzerland; Department of Psychology, University of Zurich, CH-8050 Zurich, Switzerland

**Keywords:** health-related social control, social influence strategies, health behavior change, intensive longitudinal, romantic couples

## Abstract

**Background:**

Physical activity is essential for health and wellbeing. However, many individuals fail to reach the recommended levels and obesity rates are increasing. Health-related social control refers to strategies employed by 1 person (agent) to influence another person’s (target) health behavior. These strategies can be classified into persuasion (eg, encouraging or motivating) or pressure (eg, nagging or coercing). However, much of the existing research is cross-sectional and mostly focuses on the experiences of the targets.

**Purpose:**

This study investigates how persuasion and pressure within overweight romantic couples relate to outcomes in both agents and targets. Specifically, it examines same-day associations with positive and negative affect, as well as physical activity.

**Methods:**

This study is a secondary analysis of the 14-day follow-up period from a randomized controlled trial. Accelerometers and daily diaries tracked 99 overweight romantic couples. For each outcome and each partner, separate multilevel models were fitted.

**Results:**

Daily persuasion used by agents was associated with increased physical activity in targets and a more favorable affect in agents. Daily pressure was not associated with the physical activity of either partner but was linked to a more unfavorable affect in the agent. Both persuasion and pressure were unrelated to the targets’ affect.

**Conclusions:**

Health-related social control in romantic relationships relates to same-day outcomes of both agents and targets. Our findings suggest that health behavior change interventions and weight loss programs could benefit from encouraging persuasion and limiting pressure.

## Teaser

In overweight couples, on days when one partner used persuasion, the other's physical activity was higher, and the persuader felt better. In contrast, daily pressure was not associated with physical activity, and the partner exerting pressure felt worse.

## Introduction

Physical activity is crucial for physical and mental health, as well as general wellbeing.^1^ It lowers the risk of many noncommunicable diseases like type 2 diabetes, cardiovascular disease, and stroke, and can help with weight loss.^1,2^ Despite these benefits, around 31% of adults worldwide do not meet the World Health Organization’s (WHO) recommended weekly activity levels of at least 150 minutes of moderate, or 75 minutes of vigorous physical activity.^3,4^ This contributes to the globally rising rates of obesity, with already 1 in 8 adults being classified as obese, and 43% as overweight.^5^ Understanding why people engage or do not engage in healthy behaviors, and what happens during health behavior change attempts, is important to improving health outcomes in the general population, especially among the overweight and obese.

Established health behavior models have provided important insights into intraindividual processes in health behavior change. However, interpersonal processes have been less explored, despite emerging evidence suggesting their importance. For example, many adults perceive their romantic partners as their primary source of support and influence for health behaviors.^6,7^

Romantic partners can influence each other’s health behaviors through multiple pathways, described in the dyadic health influence model.^8^ One of the main routes includes influence strategies. They refer to any intentional efforts of 1 person (the agent) to influence or regulate another person’s (the target) health behaviors. These strategies are also referred to as *health-related social control*.^9^ The original dual-effects model of social control proposes that these strategies can increase health behavior in the target while negatively impacting their affective state.^9,10^ Subsequent research has shown that different types of strategies can have different effects and began classifying them into *positive control* and *negative control*.^9,11^ In this study, we adopt an alternative terminology suggested by Stephens et al.,^12^ aiming to avoid implicit connotations associated with *positive* and *negative*, and to categorize strategies based on their characteristics, rather than assumed effects. Specifically, we will use persuasion instead of positive, and pressure in place of negative control.

Persuasion refers to social control strategies aiming to encourage the target to voluntarily engage in the behavior, for example by using positive reinforcement or reminders.^13^ Persuasion is generally associated with favorable outcomes in the target, like more positive affect (PA), less negative affect (NA), and improved health behavior.^11,14^ Many mechanisms have been proposed and they may vary between different strategies. For instance, strategies like (1) agents acting as models, (2) agents mitigating barriers, or (3) agents encouraging targets, may positively affect health beliefs, self-efficacy, and self-monitoring.^8^ In contrast, exerting pressure aims to compel the target, for example, by coercing, inducing guilt, or threat of punishment.^13^ Pressure is typically associated with unfavorable outcomes in the target: It is unrelated or even negatively related to health behavior and linked to more unfavorable affect.^11,14^ This can be explained by the intertwined model of reactance,^15,16^ which suggests that control attempts perceived as autonomy-limiting can provoke reactance, that is, a combination of anger and negative cognition motivating the target to regain their autonomy and resist the request or even do the opposite.^11^

Although social control is inherently dyadic, most research has focused only on targets. But insights from related fields suggest the need to also explore the agent’s experience. For instance, providing social support is linked to positive affective, behavioral, and relational outcomes in agents,^6^ likely due to activation of self-regulatory processes^6^ and feeling needed and valuable.^17^ Drawing parallels, utilizing persuasion might yield similar benefits by activating the agent’s own health goals and self-monitoring while priming the behavior. Agents may also feel instrumental in their partner’s health, leading to increased esteem, affect, and self-efficacy. Thus, social support and persuasion are likely related constructs that may operate through similar mechanisms. They may also frequently co-occur within the same days. Therefore, to accurately examine the unique effects of persuasion, any potential overlap with social support should be accounted for in analyses.

Conversely, the use of pressure might lead to a reactant response from the target, which, in turn, could diminish the agent’s own affect, feelings of self-efficacy, and motivation to engage in healthy behaviors. Early evidence supports the existence of links between persuasion and favorable outcomes for the agent. Lewis and Butterfield^7^ found that wives who persuaded their partners more, experienced larger positive changes in their own health behavior. The authors suggest an additional pathway: the use of strategies requiring the agent’s active involvement, such as modeling or offering joint engagement in the behaviors. Alternatively, individuals with high intentions, positive attitudes toward health behaviors, and high self-efficacy are likely to engage in more health behaviors^18^ and are simultaneously more likely to influence their partner’s health behaviors. Thus, it is possible that pathways go both ways and feedback mechanisms are at play.^8^

Past studies investigated associations of social control strategies primarily with cross-sectional designs, observing differences between individuals. However, these designs do not allow for conclusions about within-person associations.^19^ Recent research suggests that the same associations between social control and health behavior, affect, and reactance-related reactions may also occur at the within-person level, and these processes unfold over short time frames. For instance, 1 study found the expected same-day associations but no links with outcomes on subsequent days.^11^ However, to our knowledge, no study has comprehensively examined same-day or lagged relationships for both targets and agents.

The current study addresses these limitations of previous research. First, it employs an intensive longitudinal design to examine same-day associations of persuasion and pressure with physical activity and affect. Second, it investigates these associations not only for the target, but also for the agent, expanding the focus to the dyad. Notably, in this study, 1 partner was randomly selected as the target of the health behavior change intervention, while the other assumed a supportive role (referred to here as agent). Exerted social control was measured only in the agent. Thus, no reciprocal processes are analyzed.

### Hypotheses

All hypotheses were preregistered on the Open Science Framework (OSF). Based on the extended dual-effects model and previous findings,^9,11^ we expected that on days when agents use more persuasion, targets (H1) engage in more moderate to vigorous physical activity (MVPA), while (H2) experiencing more PA and (H3) less NA. Conversely, we hypothesize that on days when agents use more pressure, targets (H4) engage in less MVPA, while (H5) experiencing less PA and (H6) more NA.

Considering the potential for feedback-loop processes and direct effects of the exertion of certain control strategies on the agents themselves, we also formulated hypotheses for outcomes in the agent: On days when agents use more persuasion, agents (H7) engage in more MVPA, while (H8) experiencing more PA and (H9) less NA. And on days when agents use more pressure, agents (H10) experience less PA and (H11) more NA. Lastly, the assumed mechanisms of how persuasion affects the agent’s MVPA should not apply to pressure. Therefore, we do not expect pressure to be associated with the agent’s MVPA. However, we will investigate this relationship exploratively to account for the possibility of distinct feedback mechanisms.

## Method

This study is a secondary analysis of a single-blind randomized controlled trial aimed at increasing physical activity in overweight romantic couples. The trial began with a baseline assessment, followed the next day by a 14-day intervention and then a 14-day observation phase (28 days total). Six months after the baseline (about 5 months after the 28-day period), a 14-day follow-up assessment took place. The current analysis focuses on this 14-day follow-up. A full description of the trial and its results is provided by Berli and Scholz.^20^ This report follows the Strengthening the Reporting of Observational Studies in Epidemiology (STROBE) checklist.^21^

### Sample and design

The original study recruited *N* = 121 heterosexual couples from the Swiss population with flyers, advertisements, and the help of a market research institution, between March 2012 and October 2013. The sample size was established through a power analysis to ensure statistical power for detecting differences between the control and intervention groups. Inclusion criteria were that both partners had to be overweight or obese (body mass index [BMI] ≥25 kg/m^2^) and physically inactive but with the intention to increase their physical activity. They also had to be in a relationship for a minimum of 12 months, cohabit for at least 6 months, and both be proficient in German. Exclusion criteria were current participation in a weight loss program, working in 24-hour shift work, and being pregnant. Included couples provided written informed consent at the lab. Within each couple, 1 partner was randomly assigned the role of the primary target for the behavior change attempt, with the other assuming a supportive role (agent). Couples were randomized into 3 groups. In group 1, targets set behavioral goals alone and then received standardized daily action control text messages from study personnel over a 14-day diary period. In group 2, targets set behavioral goals together with their partners and received the same standardized texts, but from their partners. The control group did not set behavioral goals and only received neutral reminders to fill out the diaries. No differences between groups and no long-term intervention effects emerged in the initial trial.^20^

The follow-up data, on which the current study is based, were collected between October 2012 and June 2014 over a 14-day period, 6 months after baseline (about 5 months after the initial intervention and observation phase ended). No interventions were administered during this assessment. Participants wore accelerometers and completed daily diaries on their smartphones within 1 hour of bedtime and were instructed to not discuss their responses with their partners. Couples received 200 Swiss francs (CHF) after completing both the main phase and the follow-up.

A total of *N* = 99 participants (82% of the initial sample) took part in this follow-up with half (49) of the couples from the control group, 30 from group 1, and 20 from group 2. In 49 cases, the target of the behavior change was the female partner, and in 50 cases, the male partner. In the final sample, targets mean age was 46.31 years (SD = 13.68, range: 22-72) and their mean BMI was 30.94 (SD = 5.60, range: 24.98-61.73). Agents had a mean age of 46.29 years (SD = 13.72, range: 23-75) and a mean BMI of 31.24 (SD = 4.18, range: 25.18-49.61). Across targets and agents, 69% reported at least 1 health-related issue. The most common were joint or bone problems (30%) and the use of heart medications (30%). Previously, during exertion or at rest, 6% had experienced chest pain, 20% breathing difficulties, and 14% dizziness or fainting. Furthermore, 7% had diabetes, 4% had cardiovascular disease, and 10% reported other issues such as asthma, chronic pain, or back problems. To ensure the safety of the participants, individuals who reported at least 1 relevant health issue in the screening questionnaire (physical activity readiness questionnaire; PAR-Q^22^) were required to get clearance from their general practitioners prior to baseline assessment.

On average, couples had been in a relationship for 19.09 years (SD = 14.30, range = 1.08-52.00), cohabiting for 17.30 years (SD = 14.37, range 1.08 = 52.00), with 70% being married. While 57% of the couples had children, only 41% were living with at least 1 child during data collection. Overall, 24% of agents and 29% of targets had obtained tertiary-level qualifications, while 63% of agents and 64% of targets had completed vocational training. In Switzerland, the mean net income of households during the period from 2012 to 2014 was 9067 CHF.^23^ In our sample, 24% of couples reported their net household income as between 8001 and 10 000 CHF, with 51% earning less and 25% earning more.

### Measures

The variables of interest were collected on a daily level over 14 days. Table 1 lists descriptive statistics, including intraclass correlation coefficients (ICCs). Within- and between-person correlations are reported in Supplementary Material 1 on OSF.

**Table 1.** Descriptive statistics for focal variables.

	*n*	Missing	*M*	SD	Range	ICC
Persuasion agent	1386	4.40%	1.32	0.49	1.00-3.50	0.53
Pressure agent	1386	4.40%	1.09	0.27	1.00-3.25	0.48
Positive affect agent	1386	4.40%	3.83	0.89	1.00-6.00	0.55
Positive affect target	1386	5.84%	3.88	0.92	1.00-6.00	0.63
Negative affect agent	1386	4.40%	1.91	0.84	1.00-5.20	0.60
Negative affect target	1386	5.84%	1.83	0.80	1.00-5.80	0.66
MVPA agent (in minutes)^a^	1209	12.77%	47.47	36.31	0.00-239.00	0.38
MVPA target (in minutes)^a^	1219	12.05%	48.76	37.94	0.00-293.00	0.44
Provided support agent	1386	4.40%	2.53	1.31	1.00-6.00	0.52
Provided support target	1386	5.84%	2.23	1.25	1.00-6.00	0.48

Abbreviations: ICC, intraclass correlation; MVPA, moderate to vigorous physical activity (in minutes); NA, negative affect.

^a^For days with accelerometer wear time of less than 10 hours, MVPA data were considered insufficient for reliable measurement and set to NA (missing).

*Exerted pressure and persuasion* were measured only in the agent, at the end of each day, by 4 items each (adapted from^24^). The following persuasion strategies were assessed: trying to convince the partner to be physically active, emphasizing the importance of the partner’s physical activity to the agent, offering compliments to the partner for their physical activity, and suggesting engaging in physical activity together. An example item was, “To positively influence the physical activity of my partner, TODAY I emphasized how important it is to me that he/she is physically active.” Within-person reliability was marginally acceptable (ω_within_ = 0.63, 95% CI = [0.57, 0.70]), and removing any items did not substantially change the reliability. The following pressure strategies were assessed: trying to induce guilt in the partner for their lack of physical activity, making insinuations about the partner’s physical activity, withholding affection, and attempting to monitor the partner’s physical activity. An item in the pressure category was, “To positively influence the physical activity of my partner, TODAY I tried to make him/her feel guilty.” Within-person reliability was marginally acceptable (ω_within_ = 0.61, 95% CI = [0.51, 0.70]), and removing any items did not substantially change the reliability. Possible responses for all items ranged from 1 (*never today*) to 4 (*often today*). Mean scores of persuasion and pressure were generated per person and day.

*Daily MVPA* of both partners was recorded device-based using hip-worn GT3X+ accelerometers (ActiGraph, Pensacola, FL, United States). The device measured acceleration on 3 axes in counts, from which a vector magnitude count was calculated. Behavior was classified as MVPA if this value was higher than 2690 counts per minute.^25^ The number of minutes spent per day on MVPA was used as the final measure. On days with less than 10 hours of accelerometer wear time, MVPA was coded as missing (NA).^26^

*Daily PA and NA* were rated by both partners on the German short version of the Positive and Negative Affect Schedule (PANAS).^27^ Both dimensions consisted of 5 items each, ranging from 1 (*not at all true today*) to 6 (*completely true today*). An example item for PA was, “TODAY I feel stimulated,” and an example item for NA was, “TODAY I feel annoyed.” Daily mean scores for PA and NA were generated. Within-person reliability for PA and NA of both agents and targets was acceptable (all ω_within_ > 0.70).

*Covariates* in our analyses included both time-variant and time-invariant variables. These variables, along with the rationale for their inclusion, are discussed in greater detail in Supplementary Material 2. The following time-invariant covariates were assessed at baseline: gender of the target (0 for females, 1 for males); relationship duration in years; age of both partners in years; BMI of both partners in kg/m^2^; having children (0 = no, 1 = one or more); and group assignment of the initial trial, dummy coded with the control group as the reference category and 2 variables named groups 1 and 2. The following time-variant covariates were collected daily: time, ranging from 0 (diary day 1) to 13 (diary day 14); time spent together in hours; weekend (0) versus weekday (1). We also included social support (scales 1-6) as a covariate to investigate the unique effects of persuasion beyond a possible overlap with support. Targets’ and agents’ support provision was computed as the means of emotional and instrumental support, as they are highly correlated (ω_within_ = 0.86, 95% CI = [0.84, 0.87]). When modeling MVPA, we additionally included accelerometer wear time in hours.^26^

### Data analysis

The R Statistical language (v4.4.1) and R Studio (v2023.12.1) were used for the analysis. Analyses were conducted at the dyad level with 1 row in the dataset corresponding to 1 couple. A significance level of .05 was used for 2-tailed statistical tests. Multilevel modeling was used to account for the temporal dependency in the data, with repeated measures on level 1 and different couples on level 2. Within dyads, partners were distinguished by their assigned role of target or agent. Same-day associations were estimated separately for each outcome and each partner. Because pressure and persuasion were only assessed from the agent, no fully dyadic analysis approach was possible. Predictor variables and covariates were centered within persons. We controlled for the person means of the focal variables, time (diary day), and weekends versus weekdays in all models, and for accelerometer wear time when modeling MVPA. For each model, random intercepts, random slopes for persuasion and pressure, and all correlations between these random effects were included. An AR(1) covariance structure was used to account for possible autocorrelations of the residuals. All reported effects are unstandardized. Missing data were addressed under the assumption that data were missing at random (MAR). The models employed restricted maximum likelihood estimation, which is robust under the MAR assumption.^28^ In instances where full models failed to converge, we first tried replacing the AR(1) covariance structure with an unstructured one. If convergence issues persisted, we removed random effect terms, aiming to retain as many terms as possible.^29^

For PA and NA, we fit linear mixed effects models featuring a Gaussian distribution and identity link functions, employing the nlme package (v3.1.166).^30^ For MVPA, we addressed the overdispersion in the outcome by using a quasi-Poisson distribution with a logarithmic link function in the glmmTMB package (v1.1.9).^31^ To facilitate a meaningful interpretation of the coefficients, we exponentiated the fixed effects and their CIs. This way, they represent incidence rate ratios (IRRs) and can be interpreted as a multiplicative change in the outcome, per 1 unit increase of the predictor. Thus, the exponentiated CIs indicate significance if the value 1 is not included. The random effects are reported on the logarithmic scale.

To evaluate the glmmTMB models, the DHARMa package (v0.4.6) was used to simulate expected residuals and compare them to the models’ residuals.^32^ To assess the fit of nlme models, the performance package (v0.12.3) was employed.^33^ We utilized lavaan (v0.6.18)^34^ to estimate McDonald’s Omega for within-person reliability (ω_within_) through a 2-level confirmatory factor analysis.^35^ We used the wbCorr package (v0.1.22)^36^ to compute within- and between-person correlations and ICCs. ICCs quantify the proportion of variance due to differences between individuals relative to daily fluctuations within individuals. Other helper packages were used, and a full list of references is available in the full analysis script on OSF.

To assess the robustness of our results, we conducted 2 sets of sensitivity analyses. In the first, we included time-invariant covariates in all models (eg, gender, BMI, etc.). In the second set, we accounted for other social exchange processes (eg, daily and mean provided social support, etc.) to test the unique effects of social control beyond the possible overlap with other social exchange processes.

Finally, in an attempt to establish temporal order, we exploratorily investigated whether social control strategies (*T*_−1_) were associated with outcomes on the next day (*T*_0_), or whether affect and MVPA at *T*_−1_ predicted the use of social control strategies on the next day (*T*_0_).

## Results

Descriptives are reported in Table 1. ICCs indicate similar variability within- and between-person for all measures (min = 0.28, max = 0.63). In line with previous results,^11^ no lagged effects emerged (Supplementary Materials 3 and 4). The following sections report the same-day analyses (Table 2). Results for agents and targets stem from separate models but are presented and discussed together for more intuitive reading and interpretation.

**Table 2.** Multilevel model estimates: persuasion, pressure, and their associations with same-day MVPA and affect.

	Positive affect				Negative affect				MVPA			
	Agent^a^		Target^b^		Agent^a^		Target^b^		Agent^c^		Target^d^	
Fixed effects	*b*	CI_95_	*b*	CI_95_	*b*	CI_95_	*b*	CI_95_	IRR^e^	CI_95_	IRR^e^	CI_95_
Intercept	3.83^***^	[3.68, 3.98]	3.98^***^	[3.82, 4.14]	1.94^***^	[1.80, 2.07]	1.83^***^	[1.69, 1.96]	44.13^***^	[39.20, 49.67]	41.72^***^	[37.00, 47.04]
Level 1 (within-person)												
Persuasion agent	0.33^***^	[0.21, 0.45]	0.01	[−0.11, 0.13]	−0.16^**^	[−0.28, −0.04]	0.02	[−0.09, 0.12]	1.19^*^	[1.03, 1.37]	1.37^***^	[1.22, 1.54]
Pressure agent	−0.28^*^	[−0.51, −0.04]	−0.01	[−0.21, 0.20]	0.22^*^	[0.04, 0.39]	−0.09	[−0.26, 0.08]	1.08	[0.88, 1.33]	0.82	[0.67, 1.01]
Time (diary days 0-13)	−0.00	[−0.01, 0.01]	−0.01^**^	[−0.02, −0.01]	0.00	[0.00, 0.01]	0.00	[0.00, 0.01]	1.00	[0.99, 1.00]	1.00	[1.00, 1.01]
Weekend (0 = no, 1 = yes)	0.04	[−0.03, 0.12]	0.04	[−0.03, 0.11]	−0.15^***^	[−0.22, −0.09]	−0.12^***^	[−0.18, −0.06]	0.90^*^	[0.83, 0.97]	0.87^***^	[0.80, 0.94]
Accelerometer wear time									1.03^**^	[1.01, 1.06]	1.05^***^	[1.03, 1.07]
Level 2 (between-person)												
Persuasion agent	0.13	[−0.33, 0.60]	0.36	[−0.14, 0.87]	0.32	[−0.11, 0.75]	−0.36	[−0.80, 0.08]	0.75	[0.53, 1.06]	1.02	[0.72, 1.45]
Pressure agent	0.16	[−0.73, 1.06]	−0.73	[−1.70, 0.25]	0.09	[−0.73, 0.92]	0.50	[−0.34, 1.33]	1.83	[0.89, 3.75]	1.24	[0.63, 2.44]
Accelerometer wear time									0.99	[0.94, 1.05]	1.06^*^	[1.00, 1.12]

	Positive affect				Negative affect				MVPA			
	Agent^a^		Target^b^		Agent^a^		Target^b^		Agent^c^		Target^d^	
Random effects^f^	Estimate	CI_95_	Estimate	CI_95_	Estimate	CI_95_	Estimate	CI_95_	Estimate^e^	CI_95_	Estimate^e^	CI_95_
SD intercept	0.64	[0.55, 0.75]	0.72	[0.62, 0.83]	0.62	[0.54, 0.72]	0.63	[0.54, 0.73]	0.47	[0.40, 0.56]	0.43	[0.20, 0.97]
SD persuasion agent	0.24	[0.12, 0.49]	0.20	[0.08, 0.50]	0.27	[0.17, 0.44]	0.26	[0.16, 0.42]	0.27	[0.16, 0.45]	0.18	[0.06, 0.52]
SD pressure agent	0.33	[0.12, 0.92]	0.15	[0.02, 1.29]	0.23	[0.08, 0.68]	0.27	[0.10, 0.67]			0.12	[0.01, 1.02]
Additional parameters												
SD residual	0.61	[0.59, 0.64]	0.57	[0.55, 0.60]	0.54	[0.51, 0.56]	0.47	[0.45, 0.49]				
Dispersion									15.31	[13.88, 16.89]	14.40	[12.92, 16.06]
Autocorrelation (AR1)	0.19										0.97	

Abbreviation: MVPA, moderate to vigorous physical activity (minutes). Persuasion and pressure used by the agent were measured on a 4-point Likert scale, the other discrete variables on a 6-point scale. Nonbinary predictors were centered within- and between-person. Separate models were estimated per outcome and partner. A total of *N* = 99 couples were included.

Total observations:

^a^ *n* = 1325.

^b^ *n* = 1269.

^c^ *n* = 1172.

^d^ *n* = 1174.

^e^For models with a logarithmic link function, random effects are reported on the log scale, while coefficients and CIs were exponentiated, yielding incidence rate ratios (IRRs), representing multiplicative changes.

^f^The SD of the random slopes or intercepts. To achieve convergence, some estimates could not be computed.

^*^ *P* < .05.

^**^ *P* < .01.

^***^ *P* < .001.

### Daily MVPA

The intercept for MVPA was 41.27 for targets (*P* < .001, 95% CI [37.00, 47.04]) and 44.13 for the agents (*P* < .001, 95% CI [39.20, 49.67]). This reflects their average activity in minutes with all predictors at zero, that is, on the first study day with average persuasion and pressure. The SD of the random intercepts indicates variability between individuals for both partners (see Table 2).

Both hypotheses regarding persuasion and MVPA were supported. On days when agents used more persuasion, both targets (H1) and agents (H7) engaged in significantly more MVPA: For each unit increase in persuasion used by agents, the targets engaged in 37% more MVPA (IRR = 1.37, *P* < .001, 95% CI [1.22, 1.54]), and the agents engaged in 19% more MVPA (IRR = 1.19, *P* = .016, 95% CI [1.03, 1.37]). The SD of the random slopes for persuasion indicated some variability in the effect size between couples.

Our hypothesis regarding pressure and the target’s MVPA (H4) was not supported: On days when agents used more pressure, there was no significant change in the target’s MVPA (IRR = 0.82, *P* = .058, 95% CI [0.67, 1.01]). Exploratively, we also looked at the agent’s own MVPA and there was also no significant change (IRR = 1.08, *P* = .473, 95% CI [0.88, 1.33]). The random slope’s SD for pressure in targets indicated little variability in effect sizes. For agents, no random slopes could be estimated due to model convergence issues.

### Daily PA and NA

Intercepts indicated that PA on the first study day with average persuasion and pressure was 3.98 for targets (*P* < .001, 95% CI [3.82, 4.14]) and 3.83 for agents (*P* < .001, 95% CI [3.68, 3.98]). For NA, the intercepts were 1.83 for the targets (*P* < .001, 95% CI [1.69, 1.96]) and 1.94 for the agents (*P* < .001, 95% CI [1.80, 2.07]). The random intercept SDs for both PA and NA showed variability between individuals (see Table 2).

None of our hypotheses regarding the targets’ affect were supported. Contrary to our predictions, daily use of persuasion (H2 and H3) or pressure (H5 and H6) by agents was not significantly related to the targets’ PA and NA.

Both hypotheses regarding persuasion and the agents’ affect were supported: For each unit increase in persuasion used by agents, the agents’ own PA (H8) increased by 0.33 units (*b* = 0.33, *P* < .001, 95% CI [0.21, 0.45]) and their NA (H9) decreased by 0.16 units (*b* = −0.16, *P* = .008, 95% CI [−0.28, −0.04]). The SD of the random slopes for persuasion on agents’ PA and on agents’ NA indicated some variability of effect sizes between individuals.

Both hypotheses regarding pressure and the agent’s affect were also supported: For each unit increase in exerted pressure, the agents’ PA (H10) decreased by 0.28 units (*b* = −0.28, *P* = .021, 95% CI [−0.51, −0.04]) and their NA (H11) increased by 0.22 units (*b* = 0.22, *P* = .016, 95% CI [0.04, 0.39]). Finally, the random slopes for pressure in agents’ PA and NA indicated that there was some variability of effect sizes between individuals.

### Sensitivity analyses

The inclusion of time-invariant covariates (gender, relationship duration, age, BMI, having children, assignment to the initial study groups; see Supplementary Material 5), did not change the pattern of results. However, accounting for social exchange processes (daily and mean time spent together, and the agent’s and the target’s daily and mean provided support; see Supplementary Material 6) altered some of the associations. Persuasion was no longer positively related to the agent’s MVPA and no longer negatively related to the agent’s NA. Additionally, a new negative relationship between pressure and the target’s MVPA emerged, with a 22% decrease in MVPA per unit increase in pressure, on the same day (IRR = 78, *P* = .034, 95% CI [0.64, 0.96]). All other associations remained robust.

## Discussion

This study aimed to enhance our understanding of how persuasion and pressure relate to affect and physical activity in the daily life of overweight or obese romantic couples. We investigated same-day associations not only for the target of these social control strategies but also for the agent exerting control. In this study, associations of social control emerged within the same day, while exploratory lagged analyses did not reveal any links across days.

### Associations for the target

As expected, on days when agents used more persuasion the target’s physical activity was higher (H1). This finding is in line with previous research linking persuasion to improved health behavior^7,11,13,14^ and confirms this association at the within-person level in everyday life. Our sensitivity analyses establish the robustness of this result.

Conversely, we expected that pressure would be negatively related to physical activity in the target (H4).^11,14^ This was based on the intertwined model of reactance, which proposes that autonomy-limiting control strategies lead to resistance or even opposite behavior in the targets.^15,16^ However, our initial findings did not reveal a significant relationship between pressure and the target’s physical activity. As previous results have been mixed and reported effect sizes small,^14^ the absence of a significant association in our study is not entirely surprising. At the very least, our findings are in line with the notion that pressure is not an effective control strategy (see ^11^). Interestingly, when accounting for other social exchange processes in sensitivity analyses, the association between pressure and the target’s physical activity became significant and negative. Negative effects of pressure may have been buffered by compensatory positive social interactions later in the same day, possibly as part of “making up” between partners. This is supported by a significant positive within-person correlation between pressure and social support (*r* = 0.13, *P* < .001).

Regarding affect, we anticipated that daily persuasion would be associated with more PA (H2) and less NA (H3) in the target, on the same day. Conversely, we hypothesized that pressure would be linked to less PA (H5) and more NA (H6) in the target. However, persuasion and pressure were both unrelated to the target’s daily affect. These results did not change in our sensitivity analyses. This was unexpected, although previous results have been somewhat mixed.^14^ It is possible that this association is difficult to detect due to affect being a fast-changing construct that can be influenced by many events throughout a single day.^37^ Possibly, the potential effects of pressure or persuasion dissipated over the course of the day and remained undetected by our daily evening questionnaires. This is supported by Scholz et al.,^11^ who asked participants about their affective reactions to control attempts with the item, “How did you feel today after your partner tried to influence you this way?” The associations between both persuasion and pressure with this control-specific affect item were substantial. This indicates an immediate positive and negative affective response to persuasion and pressure, respectively. Future studies should consider incorporating both general and control-specific affect measurements to capture these dynamics more accurately across different time scales.

### Associations for the agent

The hypothesized positive association between the agents’ daily use of persuasion and the agent’s own MVPA on the same day (H7) was initially supported. This aligns with previous work,^7^ proposing agents might choose strategies that inadvertently impact their own behavior, such as participating in joint activities, modeling the behavior, or altering the shared environment.^7,8^ Additionally, feedback mechanisms may play a role.^8^ For example, once persuaded to be active, the target may act as a role model for the agent. The act of persuasion could also make the health behavior more salient to the agent, activate their own health goals, and promote self-monitoring. Finally, it is possible that agents use more persuasion on days when they are more active themselves. However, our sensitivity analysis revealed that this association did not persist when accounting for other positive social exchange processes. Being provided with physical activity-specific social support was more strongly associated with physical activity levels, potentially because the effect is much more direct, compared with trying to persuade another person to be physically active, which may only indirectly benefit the agent’s own physical activity. Future work should investigate the distinct effects of social support and social control strategies and their overlap further with higher powered studies.

We did not formulate a hypothesis regarding a possible relationship between pressure and the agent’s MVPA but investigated this exploratively. Our analyses revealed no significant association. Further research is needed to clarify the effects of pressure on each partner under various circumstances.

All our hypotheses regarding the agent’s use of social control and the agent’s affect received support. On days when agents used more persuasion, agents experienced more PA (H8) and less NA (H9). Conversely, increased daily use of pressure was related to less PA (H10) and more NA (H11) in the agent on the same day. Sensitivity analyses did not alter the overarching pattern of these results, although the relationship between persuasion and the agent’s NA no longer reached significance when accounting for other positive social exchange processes. The favorable associations between persuasion and the agent’s affect could be due to the agent feeling satisfaction after successfully persuading their partners. Additionally, as previously discussed, using persuasion was linked to more physical activity in the agents themselves. This increase in (joint) physical activity could also have been responsible for the agent’s enhanced daily affect. However, as our analyses were limited to same-day associations, the directionality of the effects remains uncertain. Therefore, it is equally plausible that the direction is inverse, and the association is explained by more PA in the agents increasing the likelihood of them using persuasion. Regarding the unfavorable associations between pressure and the agent’s affect, we expected that the underlying dynamics align with the literature on reactance.^15,16^ Specifically, targets might resist pressure and may even act opposite to the agent’s intended outcomes. This could lead to frustration in the agents, as they were unable to reach their goal. Furthermore, reactance may manifest itself in strong negative affective responses and could even lead to fighting. This could, in turn, negatively impact the agent’s affect. However, we previously discussed that we did not find significant associations between control strategies and the target’s affect, which we attributed to the fast-changing nature of affect. Therefore, any possible changes in the agent’s affect resulting from feedback loops involving the target’s affect should also not be detectable with the present data. One possibility is that using pressure makes agents feel bad or ashamed, independent of the reaction of the target, as this might simply not reflect their relationship norms. Alternatively, it is plausible that agents are more likely to exert pressure on days when they are already in a more negative affective state. Exerting pressure could even be a form of emotion regulation for agents.

### Strengths and limitations

This study has several strengths. First, we close research gaps by investigating associations of social control strategies not only for the target but also for the agent. Additionally, many prior studies employ cross-sectional designs, and less is known about within-person dynamics.^38^ Our study aimed to replicate the established effects of social control explicitly on the within-person level. This enables more robust conclusions about the associations between different control strategies and their outcomes. Another strength of our study is that our sample consists of overweight and obese couples. This population is at an increased risk for various diseases and early mortality^5^ and research promoting health behavior change in these groups is especially relevant because it can help mitigate these risks.^39,40^ Finally, by employing hip-worn accelerometers to measure physical activity, we avoid biases inherent to self-report measures and address the issue of shared method variance for this outcome. This increases the validity of our findings. Besides its strengths, our study also has several limitations. First, the marginally acceptable within-person reliability for persuasion and pressure may affect the precision of our analyses. This likely reflects that specific control strategies are distinct and do not always occur together, even though they are in the same category. Thus, our measurement model may be additive (formative) instead of reflective, making internal consistency less crucial for validity.^41^ Analyzing individual strategies could be a viable alternative to pooling, but their infrequent use combined with our small sample limited our ability to do so, as well as our study’s power. Future studies should use larger samples or longer time frames to explore individual strategies and develop more precise general measures of social control. Another limitation was that our design does not allow for conclusions about causality or the directionality of effects, due to the study’s design. Future research should employ experimental designs to explore causal relationships. Additionally, 1 measurement per day may not have been enough to capture some of the faster-changing constructs, like affect, adequately. Future studies could benefit from investigating dynamics on multiple time scales. Another limitation is that some constructs were measured via self-report, possibly introducing measurement errors. While our use of daily diaries mitigates recall bias by minimizing the intervals between events and reports,^42^ social desirability could have led to underreporting of exerted control, especially pressure.^43^ Although focusing on overweight and obese couples with various health conditions is a strength of our study, it does limit the generalizability of our results. While we do not anticipate fundamental differences in underlying mechanisms, variations may still exist. For instance, in populations like the one in our study, agents and targets may both perceive a greater threat to the target’s health by not being physically active. This could increase the likelihood of agents attempting to influence their partners’ behaviors. Additionally, targets could perceive control strategies as more acceptable.^8^ Additionally, the current study does not directly investigate which mechanisms drive the effects of social control for both agents and targets. Future studies should address this by investigating both individual and social processes and their interplay. Finally, although the current study examines outcomes for both partners, only 1 partner per couple was assigned the role of the target, while the other was the agent. This may limit the generalizability of our results to such unbalanced dyads. Future work should measure the use of pressure and persuasion of both partners and employ fully dyadic designs to obtain a better understanding of these dyadic dynamics from all points of view.

## Conclusion

This study highlights the important role of health-related social control strategies within obese and overweight romantic couples. We address research gaps and limitations of previous studies by exploring within-person associations not only for targets, but also agents. The following results remained robust in sensitivity analyses: The agent’s persuasion was associated with higher physical activity in targets. Exerted pressure was unrelated to physical activity in both partners. Both persuasion and pressure were unrelated to the targets’ affect. However, the agents’ affect was more favorable on days when they tried to persuade their partners more, and less favorable on days when they exerted more pressure. Our findings demonstrate the importance of considering both partners’ perspectives. These insights may inform the development of future health behavior change interventions and weight loss programs: Including romantic partners and educating them on persuasion and pressure could improve health behaviors in both partners, while avoiding negative outcomes. The literature would greatly benefit from more longitudinal or experimental dyadic studies with both partners being agents and targets simultaneously, enabling more comprehensive analyses of processes within couples.

## Supplementary Material

kaae093_suppl_Supplementary_Material
