# Supplementary material for: Health-related social control in overweight romantic couples: daily associations with physical activity and affect for targets and agents
Source: Ann Behav Med. 2025 Jan 8;59(1):kaae093. doi: 10.1093/abm/kaae093 (PMC11707529; doi:10.1093/abm/kaae093)
Supplement: kaae093_suppl_Supplementary_Material [file kaae093_suppl_supplementary_material.docx]

# Electronic Supplementary Material

**Electronic Supplementary Material 1**

*Pearson Correlations Within and Between Persons and Intraclass Correlations*

|  |  | 1 | 2 | 3 | 4 | 5 | 6 | 7 | 8 | 9 | 10 | 11 | 12 | 13 | 14 | 15 |
| --- | --- | --- | --- | --- | --- | --- | --- | --- | --- | --- | --- | --- | --- | --- | --- | --- |
| 1 | Persuasion agent | **[0.53]** | 0.36*** | 0.17*** | 0.02 | -0.10*** | -0.03 | 0.15*** | 0.09** | 0.44*** | 0.26*** | -0.10*** | -0.10*** | 0.24*** | -0.06* | 0.18*** |
| 2 | Pressure agent | 0.61*** | **[0.48]** | -0.01 | -0.01 | 0.04 | -0.04 | 0.06* | -0.03 | 0.13*** | 0.02 | -0.09** | -0.06* | 0.02 | 0.02 | 0.04 |
| 3 | Positive affect agent | 0.07 | 0.05 | **[0.55]** | 0.16*** | -0.35*** | -0.07* | 0.12*** | 0.05 | 0.16*** | 0.08** | 0.10*** | -0.02 | 0.10*** | -0.04 | 0.05* |
| 4 | Positive affect target | 0.04 | -0.08 | 0.24* | **[0.63]** | -0.12*** | -0.32*** | 0.04 | 0.10*** | 0.05 | 0.10*** | -0.04 | 0.08** | 0.03 | -0.10*** | 0.05 |
| 5 | Negative affect agent | 0.21* | 0.13 | -0.29** | -0.16 | **[0.60]** | 0.19*** | -0.10*** | 0.01 | -0.13*** | -0.10*** | -0.00 | 0.05 | -0.17*** | 0.04 | -0.15*** |
| 6 | Negative affect target | -0.08 | 0.02 | -0.21* | -0.33*** | 0.22* | **[0.66]** | 0.02 | -0.06 | -0.06* | -0.11*** | 0.03 | -0.01 | -0.04 | 0.03 | -0.11*** |
| 7 | MVPA agent (in minutes) | 0.05 | 0.20* | 0.04 | -0.12 | 0.04 | -0.04 | **[0.38]** | 0.29*** | 0.15*** | 0.19*** | 0.10*** | 0.05 | -0.02 | -0.03 | 0.01 |
| 8 | MVPA target (in minutes) | 0.06 | 0.06 | 0.04 | 0.01 | 0.25* | -0.16 | 0.50*** | **[0.44]** | 0.16*** | 0.15*** | 0.08** | 0.12*** | -0.05 | 0.04 | -0.09** |
| 9 | Provided support agent | 0.55*** | 0.22* | -0.03 | -0.12 | 0.34*** | 0.08 | 0.01 | 0.10 | **[0.52]** | 0.39*** | -0.11*** | -0.10*** | 0.19*** | -0.06* | 0.16*** |
| 10 | Provided support target | 0.32** | 0.11 | 0.08 | 0.20* | -0.00 | 0.19 | -0.01 | -0.07 | 0.45*** | **[0.48]** | -0.10*** | -0.03 | 0.24*** | -0.10*** | 0.19*** |
| 11 | Wear time agent (in minutes) | -0.11 | -0.29** | 0.25* | 0.29** | -0.06 | -0.08 | -0.14 | -0.05 | 0.02 | 0.04 | **[0.34]** | 0.29*** | -0.17*** | -0.06* | -0.24*** |
| 12 | Wear time target (in minutes) | -0.08 | -0.15 | 0.10 | 0.38*** | -0.00 | -0.07 | -0.23* | 0.06 | 0.01 | -0.00 | 0.47*** | **[0.31]** | -0.25*** | -0.08** | -0.25*** |
| 13 | Time spent together (in minutes) | 0.00 | 0.03 | 0.16 | 0.09 | -0.23* | -0.11 | -0.02 | 0.05 | 0.05 | 0.18 | 0.17 | 0.25* | **[0.29]** | 0.02 | 0.48*** |
| 14 | Time (diary day 0 - 13) | NA | NA | NA | NA | NA | NA | NA | NA | NA | NA | NA | NA | NA | **[0.00]** | -0.06* |
| 15 | Weekend (no = 0; yes = 1) | 0.18 | -0.03 | 0.16 | 0.19 | -0.11 | -0.09 | 0.06 | 0.05 | 0.15 | 0.09 | 0.12 | 0.12 | 0.20* | NA | **[0.00]** |

*Note*. MVPA = Moderate to vigorous physical activity.

Within-person correlations are displayed above the diagonal in the upper right triangle, and between-person correlations are shown below the diagonal. Intraclass correlations (ICCs), representing the proportion of variance attributed to between-person differences, are noted on the diagonal, enclosed in square brackets. This analysis was conducted using the R package wbCorr [1] and involved partitioning the variance of each variable into within- and between-person components. For a comprehensive table that includes all covariates, refer to the Supplementary Table 2.

**p* < 0.05. ***p* < 0.01. ****p* < 0.001.

**Electronic Supplementary Material 2**

*Description of and Background for Included Covariates.*

In our analysis both time-invariant and time-variant covariates were included. The following time-invariant covariates were assessed at baseline: *Gender of the target* was included to account for gender differences in physical activity [2], the use of social control [3], affective responses when being controlled [4], and effectiveness of social control strategies [5,6]. Since our sample comprised only heterosexual couples, we included only the target’s gender due to the gender of the agent and target being perfectly correlated. Gender was coded as 0 for females and 1 for males. *Relationship duration:* Introduced to account for varying dynamics of couple interaction and receptivity to social control in different phases of their relationships [7]. The variable represents relationship length in years. *Age of both partners* was included to account for age-related differences in emotion regulation and mood [8], which could reflect in the type of control strategies used and, in the reaction, when experiencing social control. The age of both partners is expressed in years. *Body Mass Index (BMI) of both partners* was accounted for, as individuals with higher BMI may experience more frequent control strategies, and they might react more adversely to these strategies [9]. To control for this, we include BMI (kg/m^2^) for both partners. *Having children* was taken into account, as this could decrease the amount of time available for physical activity [10] and change the occurrence of social control interactions. Parental status was thus considered as a binary variable, with 0 indicating no children and 1 indicating one or more children. *Group assignment* was included to control for any residual or lasting impacts from the initial intervention study, although no long-term intervention effects at follow-up were previously found for MVPA [11]. The three groups of the study were dummy coded with the control group acting as the reference category, and a variable introduced for group 1 (individual action control group), and group 2 (dyadic action control group).

The following time-variant covariates were collected dfaily: *Time* controls for possible time trends and was included in all models, following recommendations of Bolger and Laurenceau [12]. The variable ranges from 0 (diary day 1) to 13 (diary day 14). *Daily time spent together* accounts for the influence of shared time during waking hours on opportunities for social control and collaborative activities and other social exchange processes occurring during this time. This variable is computed by taking the mean of both partners’ report. The final measure is in hours per day. *Weekend vs. weekday* takes routines and opportunities for physical activity and social control into account, which often differ between weekdays and weekends [13]. This covariate was coded 0 for weekdays and 1 for weekends. *Daily social support provision by both actor and target* can have effects on affect and physical activity [14,15], and there may be some overlap with social control. It was measured using two items, one for emotional and one for instrumental support, which were adapted from Bolger et al. [16]. Both items were formulated to specifically capture social support related to physical activity. The mean of both items was used due to their high intercorrelation [15]. The final construct is represented on a scale from 1 to 6. When modelling MVPA we included *accelerometer wear time* to account for different wear patterns. For this purpose, wear time was computed following the algorithm by Choi et al. [17] in hours per day.

**Electronic Supplementary Material 3**

Explorative Analysis for Multilevel Model Estimates: Lagged Analysis of Social Control Strategies at T_0_ predicting Outcomes at T_+1_.

|  | Next Day Positive Affect | | | | |  | Next Day Negative Affect | | | | |  | Next Day MVPA | | | | |
| --- | --- | --- | --- | --- | --- | --- | --- | --- | --- | --- | --- | --- | --- | --- | --- | --- | --- |
|  | Agent*^a^* | |  | Target*^b^* | |  | Agent*^a^* | |  | Target*^b^* | |  | Agent*^c^* | |  | Target*^d^* | |
| **Fixed Effects** | *b* | CI_95_ |  | *b* | CI_95_ |  | *b* | CI_95_ |  | *b* | CI_95_ |  | IRR*^e^* | CI_95_ |  | IRR*^e^* | CI_95_ |
| Intercept | 2.02*** | [ 1.81, 2.24] |  | 2.65*** | [ 2.41, 2.89] |  | 1.44*** | [ 1.28, 1.60] |  | 1.44*** | [ 1.28, 1.60] |  | 41.42*** | [36.12, 47.50] |  | 41.85*** | [36.59, 47.86] |
| *Level 1 (within-person)* |  |  |  |  |  |  |  |  |  |  |  |  |  |  |  |  |  |
| Persuasion agent | -0.06 | [-0.18, 0.06] |  | -0.08 | [-0.20, 0.05] |  | 0.01 | [-0.12, 0.14] |  | -0.02 | [-0.11, 0.08] |  | 1.08 | [ 0.96, 1.22] |  | 1.04 | [ 0.93, 1.17] |
| Pressure agent | 0.02 | [-0.18, 0.22] |  | -0.06 | [-0.28, 0.16] |  | -0.01 | [-0.19, 0.18] |  | 0.10 | [-0.06, 0.26] |  | 0.94 | [ 0.76, 1.17] |  | 0.86 | [ 0.70, 1.05] |
| Time (day 0 - 13) | -0.00 | [-0.01, 0.00] |  | -0.01** | [-0.02, -0.01] |  | 0.01 | [ 0.00, 0.01] |  | 0.00 | [ 0.00, 0.01] |  | 1.00 | [ 0.99, 1.01] |  | 1.01 | [ 1.00, 1.02] |
| Weekend (0 = no, 1 = yes) | 0.04 | [-0.04, 0.11] |  | 0.00 | [-0.07, 0.08] |  | -0.01 | [-0.08, 0.06] |  | -0.02 | [-0.09, 0.04] |  | 1.10* | [ 1.00, 1.20] |  | 0.96 | [ 0.88, 1.04] |
| Accelerometer wear time |  |  |  |  |  |  |  |  |  |  |  |  | 0.99 | [ 0.97, 1.02] |  | 0.97* | [ 0.95, 0.99] |
| *Level 2 (between-person)* |  |  |  |  |  |  |  |  |  |  |  |  |  |  |  |  |  |
| Persuasion agent | 0.08 | [-0.16, 0.32] |  | 0.34* | [ 0.01, 0.67] |  | 0.30 | [-0.05, 0.65] |  | -0.28 | [-0.65, 0.09] |  | 0.74 | [ 0.53, 1.05] |  | 1.04 | [ 0.74, 1.46] |
| Pressure agent | 0.04 | [-0.42, 0.51] |  | -0.54 | [-1.18, 0.09] |  | -0.03 | [-0.70, 0.64] |  | 0.36 | [-0.35, 1.06] |  | 1.75 | [ 0.84, 3.62] |  | 1.19 | [ 0.61, 2.33] |
| Accelerometer wear time |  |  |  |  |  |  |  |  |  |  |  |  | 0.98 | [ 0.93, 1.04] |  | 1.00 | [ 0.95, 1.07] |
| *Previous-day Outcome* |  |  |  |  |  |  |  |  |  |  |  |  |  |  |  |  |  |
| Positive affect agent | 0.48*** | [ 0.43, 0.53] |  |  |  |  |  |  |  |  |  |  |  |  |  |  |  |
| Positive affect target |  |  |  | 0.35*** | [ 0.29, 0.40] |  |  |  |  |  |  |  |  |  |  |  |  |
| Negative affect agent |  |  |  |  |  |  | 0.22*** | [ 0.17, 0.28] |  |  |  |  |  |  |  |  |  |
| Negative affect target |  |  |  |  |  |  |  |  |  | 0.19*** | [ 0.13, 0.25] |  |  |  |  |  |  |
| MVPA agent |  |  |  |  |  |  |  |  |  |  |  |  | 1.00 | [ 1.00, 1.00] |  |  |  |
| MVPA target |  |  |  |  |  |  |  |  |  |  |  |  |  |  |  | 1.00 | [ 1.00, 1.00] |

|  | Next Day Positive Affect | | | | |  | Next Day Negative Affect | | | | |  | Next Day MVPA | | | | |
| --- | --- | --- | --- | --- | --- | --- | --- | --- | --- | --- | --- | --- | --- | --- | --- | --- | --- |
|  | Agent*^a^* | |  | Target*^b^* | |  | Agent*^a^* | |  | Target*^b^* | |  | Agent*^c^* | |  | Target*^d^* | |
| **Random Effects*^f^*** | *Estimate* | CI_95_ |  | *Estimate* | CI_95_ |  | *Estimate* | CI_95_ |  | *Estimate* | CI_95_ |  | Estimate*^e^* | CI_95_ |  | Estimate*^e^* | CI_95_ |
| *SD* intercept | 0.30 | [ 0.22, 0.43] |  | 0.43 | [ 0.33, 0.56] |  | 0.46 | [ 0.38, 0.56] |  | 0.49 | [ 0.41, 0.59] |  | 0.44 | [ 0.37, 0.54] |  | 0.44 | [ 0.36, 0.53] |
| *SD* persuasion agent |  |  |  | 0.24 | [ 0.12, 0.50] |  | 0.30 | [ 0.18, 0.51] |  |  |  |  |  |  |  |  |  |
| *SD* pressure agent |  |  |  | 0.19 | [ 0.03, 1.29] |  | 0.15 | [ 0.03, 0.84] |  |  |  |  |  |  |  |  |  |
| Cor (intercept, persuasion) |  |  |  | 0.08 | [-0.65, 0.74] |  | 0.02 | [-0.23, 0.28] |  |  |  |  |  |  |  |  |  |
| Cor (intercept, pressure) |  |  |  | 0.21 | [-0.93, 0.97] |  | 0.24 | [-0.90, 0.96] |  |  |  |  |  |  |  |  |  |
| Cor (persuasion, pressure) |  |  |  | 0.89 | [-1.00, 1.00] |  | -0.94 | [-1.00, 0.93] |  |  |  |  |  |  |  |  |  |
| **Additional Parameters** |  |  |  |  |  |  |  |  |  |  |  |  |  |  |  |  |  |
| *SD* residual | 0.66 | [ 0.62, 0.70] |  | 0.55 | [ 0.53, 0.58] |  | 0.55 | [ 0.52, 0.57] |  | 0.48 | [ 0.46, 0.50] |  |  |  |  |  |  |
| Dispersion |  |  |  |  |  |  |  |  |  |  |  |  | 16.99 | [15.30, 18.86] |  | 16.46 | [14.83, 18.27] |
| Autocorrelation (AR1) | -0.25 | [-0.35, -0.15] |  | -0.07 | [-0.19, 0.05] |  |  |  |  |  |  |  |  |  |  |  |  |

*Note.* MVPA = moderate to vigorous physical activity (minutes). Persuasion and pressure used by the agent were measured on a 4-point Likert scale, the other discrete variables on a 6-point scale. Outcomes were lagged forward by one day. The previous day’s outcome was included as a predictor. All other non-binary predictors were centered within- and between-person. Separate models were estimated per outcome and partner. A total of *N* = 99 couples were included. Total observations: *^a^* *n* = 1324. *^b^* *n* = 1268. *^c^* *n* = 1171 *^d^* *n* = 1173. *^e^* For models with a logarithmic link function, random effects are reported on the log scale, while coefficients and confidence intervals were exponentiated, yielding incidence rate ratios (IRRs), representing multiplicative changes. *^f^* *SD* represents the standard deviation of the random slopes or intercepts. To achieve convergence, some estimates could not be computed.

**p* < 0.05. ***p* < 0.01. ****p* < 0.001.

**Electronic Supplementary Material 4**

Explorative Analysis for Multilevel Model Estimates: Inverted Lagged Analysis of Both Partners’ MVPA, PA, and NA at T_0_ predicting Persuasion and Pressure at T_+1_.

|  | Next Day Persuasion | |  | Next Day Pressure | |
| --- | --- | --- | --- | --- | --- |
| **Fixed Effects** | *b* | CI_95_ |  | *b* | CI_95_ |
| Intercept | 0.82*** | [ 0.73, 0.92] |  | 1.05*** | [ 0.97, 1.12] |
| *Level 1 (within-person)* |  |  |  |  |  |
| MVPA agent | -0.00 | [ 0.00, 0.00] |  | 0.00 | [ 0.00, 0.00] |
| MVPA target | -0.00 | [ 0.00, 0.00] |  | 0.00 | [ 0.00, 0.00] |
| Positive affect agent | -0.03 | [-0.07, 0.02] |  | -0.00 | [-0.02, 0.02] |
| Positive affect target | 0.03 | [-0.02, 0.07] |  | 0.02 | [ 0.00, 0.04] |
| Negative affect agent | 0.01 | [-0.04, 0.06] |  | -0.01 | [-0.03, 0.01] |
| Negative affect target | 0.02 | [-0.03, 0.07] |  | 0.02 | [-0.01, 0.04] |
| *Level 2 (between-person)* |  |  |  |  |  |
| MVPA agent | 0.00 | [ 0.00, 0.00] |  | 0.00 | [ 0.00, 0.00] |
| MVPA target | -0.00 | [ 0.00, 0.00] |  | -0.00 | [ 0.00, 0.00] |
| Positive affect agent | 0.04 | [-0.03, 0.10] |  | 0.04 | [-0.02, 0.09] |
| Positive affect target | 0.02 | [-0.05, 0.08] |  | -0.00 | [-0.05, 0.05] |
| Negative affect agent | 0.09* | [ 0.02, 0.17] |  | 0.05 | [-0.01, 0.12] |
| Negative affect target | -0.03 | [-0.10, 0.05] |  | 0.01 | [-0.05, 0.07] |
| *Previous-day Outcome* |  |  |  |  |  |
| Persuasion agent | 0.38*** | [ 0.32, 0.45] |  |  |  |
| Pressure agent |  |  |  | 0.04 | [-0.02, 0.10] |

|  | Next Day Persuasion | |  | Next Day Pressure | |
| --- | --- | --- | --- | --- | --- |
| **Random Effects*^f^*** | *Estimate* | CI_95_ |  | *Estimate* | CI_95_ |
| *SD* intercept | 0.19 | [ 0.14, 0.25] |  | 0.17 | [ 0.14, 0.21] |
| **Additional Parameters** |  |  |  |  |  |
| *SD* residual | 0.36 | [ 0.34, 0.38] |  | 0.16 | [ 0.15, 0.17] |
| Autocorrelation (AR1) | -0.19 | [-0.29, -0.10] |  | -0.07 | [-0.19, 0.04] |

*Note.* MVPA = moderate to vigorous physical activity (minutes). PA = positive affect. NA = negative affect. Exerted persuasion and pressure were measured on a 4-point Likert scale, other discrete variables on a 6-point scale. MVPA and affect of both partners were lagged forward by one day. The previous day’s outcome was included as a predictor. All other non-binary predictors were centered within- and between-person. Separate models were estimated per outcome and partner. A total of *N* = 99 couples were included. Total observations: *^a^* *n* = 1324. *^b^* *n* = 1268. *^c^* *n* = 1171 *^d^* *n* = 1173. *^e^* *^f^* *SD* represents the standard deviation of the random slopes or intercepts. To achieve convergence, some estimates could not be computed.

**p* < 0.05. ***p* < 0.01. ****p* < 0.001.

**Electronic Supplementary Material 5**

Sensitivity Analysis for Multilevel Model Estimates: Time-Invariant Covariates

|  | Positive Affect | | | | |  | Negative Affect | | | | |  | MVPA | | | | |
| --- | --- | --- | --- | --- | --- | --- | --- | --- | --- | --- | --- | --- | --- | --- | --- | --- | --- |
|  | Agent*^a^* | |  | Target*^b^* | |  | Agent*^a^* | |  | Target*^b^* | |  | Agent*^c^* | |  | Target*^d^* | |
| **Fixed Effects** | *b* | CI_95_ |  | *b* | CI_95_ |  | *b* | CI_95_ |  | *b* | CI_95_ |  | IRR*^e^* | CI_95_ |  | IRR*^e^* | CI_95_ |
| Intercept | 5.26*** | [ 3.90, 6.63] |  | 4.35*** | [ 2.78, 5.93] |  | 2.34*** | [ 1.08, 3.60] |  | 1.66* | [ 0.29, 3.04] |  | 126.64*** | [45.74, 350.61] |  | 234.41*** | [88.39, 621.67] |
| *Level 1 (within-person)* |  |  |  |  |  |  |  |  |  |  |  |  |  |  |  |  |  |
| Persuasion agent | 0.33*** | [ 0.20, 0.45] |  | 0.01 | [-0.10, 0.13] |  | -0.16** | [-0.28, -0.04] |  | 0.01 | [-0.09, 0.12] |  | 1.20* | [ 1.04, 1.38] |  | 1.36*** | [ 1.21, 1.52] |
| Pressure agent | -0.26* | [-0.50, -0.03] |  | -0.01 | [-0.21, 0.20] |  | 0.21* | [ 0.03, 0.38] |  | -0.10 | [-0.26, 0.07] |  | 1.08 | [ 0.88, 1.32] |  | 0.81 | [ 0.67, 1.00] |
| Time (day 0 - 13) | -0.00 | [-0.01, 0.01] |  | -0.01*** | [-0.02, -0.01] |  | 0.00 | [ 0.00, 0.01] |  | 0.00 | [ 0.00, 0.01] |  | 1.00 | [ 0.99, 1.00] |  | 1.00 | [ 1.00, 1.01] |
| Weekend (0 = no, 1 = yes) | 0.04 | [-0.03, 0.12] |  | 0.04 | [-0.03, 0.11] |  | -0.15*** | [-0.22, -0.09] |  | -0.12*** | [-0.18, -0.06] |  | 0.89** | [ 0.82, 0.97] |  | 0.86*** | [ 0.80, 0.93] |
| Accelerometer wear time |  |  |  |  |  |  |  |  |  |  |  |  | 1.03** | [ 1.01, 1.05] |  | 1.05*** | [ 1.03, 1.07] |
| *Level 2 (between-person)* |  |  |  |  |  |  |  |  |  |  |  |  |  |  |  |  |  |
| Persuasion agent | 0.22 | [-0.26, 0.71] |  | 0.42 | [-0.14, 0.97] |  | 0.35 | [-0.10, 0.80] |  | -0.47 | [-0.95, 0.02] |  | 0.92 | [ 0.65, 1.30] |  | 1.23 | [ 0.89, 1.70] |
| Pressure agent | -0.04 | [-0.95, 0.87] |  | -0.93 | [-1.96, 0.11] |  | 0.04 | [-0.80, 0.88] |  | 0.76 | [-0.16, 1.68] |  | 1.33 | [ 0.66, 2.67] |  | 0.80 | [ 0.43, 1.50] |
| Accelerometer wear time |  |  |  |  |  |  |  |  |  |  |  |  | 1.00 | [ 0.95, 1.06] |  | 1.07* | [ 1.01, 1.12] |
| Relationship length | -0.00 | [-0.02, 0.01] |  | 0.00 | [-0.01, 0.02] |  | -0.00 | [-0.02, 0.01] |  | -0.00 | [-0.02, 0.02] |  | 1.00 | [ 0.99, 1.02] |  | 1.00 | [ 0.99, 1.01] |
| Age agent | 0.01 | [-0.02, 0.04] |  | 0.01 | [-0.02, 0.05] |  | -0.03 | [-0.06, 0.00] |  | 0.01 | [-0.02, 0.04] |  | 0.99 | [ 0.97, 1.01] |  | 0.98 | [ 0.96, 1.00] |
| Age target | -0.00 | [-0.03, 0.02] |  | -0.02 | [-0.05, 0.01] |  | 0.02 | [ 0.00, 0.05] |  | -0.01 | [-0.04, 0.02] |  | 1.00 | [ 0.98, 1.02] |  | 1.00 | [ 0.99, 1.02] |
| BMI agent | -0.03 | [-0.06, 0.01] |  | -0.02 | [-0.06, 0.02] |  | -0.01 | [-0.04, 0.02] |  | 0.00 | [-0.03, 0.04] |  | 1.01 | [ 0.98, 1.03] |  | 0.98 | [ 0.96, 1.00] |
| BMI target | -0.03* | [-0.06, -0.01] |  | 0.01 | [-0.02, 0.04] |  | 0.01 | [-0.01, 0.03] |  | 0.01 | [-0.02, 0.03] |  | 0.98 | [ 0.96, 1.00] |  | 0.99 | [ 0.97, 1.00] |
| Children (No = 0, Yes = 1) | 0.03 | [-0.24, 0.31] |  | -0.03 | [-0.34, 0.29] |  | -0.01 | [-0.27, 0.25] |  | -0.05 | [-0.33, 0.22] |  | 1.21 | [ 0.99, 1.47] |  | 1.13 | [ 0.93, 1.36] |
| Gender target (F = 0, M = 1) | 0.33* | [ 0.05, 0.61] |  | 0.08 | [-0.24, 0.40] |  | -0.04 | [-0.30, 0.22] |  | -0.23 | [-0.51, 0.05] |  | 1.01 | [ 0.82, 1.23] |  | 1.07 | [ 0.88, 1.29] |
| Gender agent (F = 0, M = 1) |  |  |  |  |  |  |  |  |  |  |  |  |  |  |  |  |  |
| Dyadic Action Control | 0.09 | [-0.25, 0.44] |  | 0.11 | [-0.29, 0.52] |  | 0.26 | [-0.06, 0.58] |  | -0.06 | [-0.41, 0.29] |  | 1.04 | [ 0.81, 1.34] |  | 1.04 | [ 0.82, 1.32] |
| Individual Action Control | 0.18 | [-0.12, 0.48] |  | 0.44* | [ 0.09, 0.79] |  | -0.35* | [-0.64, -0.07] |  | -0.26 | [-0.57, 0.04] |  | 0.91 | [ 0.73, 1.13] |  | 0.91 | [ 0.74, 1.12] |

|  | Positive Affect | | | | |  | Negative Affect | | | | |  | MVPA | | | | |
| --- | --- | --- | --- | --- | --- | --- | --- | --- | --- | --- | --- | --- | --- | --- | --- | --- | --- |
|  | Agent*^a^* | |  | Target*^b^* | |  | Agent*^a^* | |  | Target*^b^* | |  | Agent*^c^* | |  | Target*^d^* | |
| **Random Effects*^f^*** | *Estimate* | CI_95_ |  | *Estimate* | CI_95_ |  | *Estimate* | CI_95_ |  | *Estimate* | CI_95_ |  | Estimate*^e^* | CI_95_ |  | Estimate*^e^* | CI_95_ |
| *SD* intercept | 0.61 | [ 0.52, 0.72] |  | 0.72 | [ 0.62, 0.85] |  | 0.60 | [ 0.51, 0.70] |  | 0.62 |  |  | 0.42 | [ 0.35, 0.50] |  | 0.36 | [ 0.15, 0.85] |
| *SD* persuasion agent | 0.25 | [ 0.13, 0.51] |  | 0.21 | [ 0.08, 0.51] |  | 0.28 | [ 0.17, 0.45] |  | 0.26 |  |  | 0.27 | [ 0.16, 0.46] |  | 0.16 | [ 0.04, 0.60] |
| *SD* pressure agent | 0.34 | [ 0.13, 0.88] |  | 0.18 | [ 0.03, 0.92] |  | 0.25 | [ 0.09, 0.68] |  | 0.22 |  |  |  |  |  | 0.10 | [ 0.03, 0.37] |
| Cor (intercept, persuasion) | -0.54 | [-0.88, 0.16] |  | -0.41 | [-0.84, 0.33] |  | -0.69 | [-0.92, -0.10] |  | -0.31 |  |  | 0.24 | [-0.29, 0.62] |  | -0.59 | [-0.92, 0.66] |
| Cor (intercept, pressure) | 0.38 | [-0.71, 0.93] |  | -0.82 | [-1.00, 0.96] |  | 0.67 | [-0.82, 0.99] |  | 0.05 |  |  |  |  |  | -0.97 |  |
| Cor (persuasion, pressure) | 0.38 | [-0.91, 0.98] |  | 0.63 | [-0.99, 1.00] |  | -0.95 | [-1.00, 0.87] |  | NA |  |  |  |  |  | 0.39 |  |
| **Additional Parameters** |  |  |  |  |  |  |  |  |  |  |  |  |  |  |  |  |  |
| *SD* residual | 0.61 | [ 0.59, 0.64] |  | 0.57 | [ 0.55, 0.60] |  | 0.54 | [ 0.51, 0.56] |  | 0.47 |  |  |  |  |  |  |  |
| Dispersion |  |  |  |  |  |  |  |  |  |  |  |  | 15.29 | [13.86, 16.87] |  | 14.41 | [12.93, 16.05] |
| Autocorrelation (AR1) | 0.19 | [ 0.13, 0.25] |  |  |  |  |  |  |  |  |  |  |  |  |  | 0.96 |  |

*Note.* MVPA = moderate to vigorous physical activity (minutes). Persuasion and pressure used by the agent were measured on a 4-point Likert scale, the other discrete variables on a 6-point scale. Non-binary predictors were centered within- and between-person. Separate models were estimated per outcome and partner. The pattern of results did not change compared to the main analysis. A total of *N* = 99 couples were included. Total observations: *^a^* *n* = 1325. *^b^* *n* = 1269. *^c^* *n* = 1172 *^d^* *n* = 1174. *^e^* For models with a logarithmic link function, random effects are reported on the log scale, while coefficients and confidence intervals were exponentiated, yielding incidence rate ratios (IRRs), representing multiplicative changes. *^f^* *SD* represents the standard deviation of the random slopes or intercepts. To achieve convergence, some estimates could not be computed.

**p* < 0.05. ***p* < 0.01. ****p* < 0.001.

**Electronic Supplementary Material 6**

Sensitivity Analysis for Multilevel Model Estimates: Additional Social Exchange Processes.

|  | Positive Affect | | | | |  | Negative Affect | | | | |  | MVPA | | | | |
| --- | --- | --- | --- | --- | --- | --- | --- | --- | --- | --- | --- | --- | --- | --- | --- | --- | --- |
|  | Agent*^a^* | |  | Target*^b^* | |  | Agent*^a^* | |  | Target*^b^* | |  | Agent*^c^* | |  | Target*^d^* | |
| **Fixed Effects** | *b* | CI_95_ |  | *b* | CI_95_ |  | *b* | CI_95_ |  | *b* | CI_95_ |  | IRR*^e^* | CI_95_ |  | IRR*^e^* | CI_95_ |
| Intercept | 3.85*** | [ 3.70, 4.00] |  | 3.98*** | [ 3.83, 4.14] |  | 1.91*** | [ 1.77, 2.04] |  | 1.83*** | [ 1.69, 1.96] |  | 43.50*** | [38.59, 49.02] |  | 40.52*** | [35.54, 46.19] |
| *Level 1 (within-person)* |  |  |  |  |  |  |  |  |  |  |  |  |  |  |  |  |  |
| Persuasion agent | 0.26*** | [ 0.12, 0.40] |  | -0.04 | [-0.16, 0.09] |  | -0.07 | [-0.20, 0.06] |  | 0.05 | [-0.06, 0.17] |  | 1.07 | [ 0.92, 1.25] |  | 1.22** | [ 1.07, 1.40] |
| Pressure agent | -0.25* | [-0.48, -0.02] |  | 0.00 | [-0.20, 0.20] |  | 0.18* | [ 0.00, 0.36] |  | -0.11 | [-0.28, 0.06] |  | 1.11 | [ 0.89, 1.38] |  | 0.78* | [ 0.64, 0.96] |
| Time (day 0 - 13) | -0.00 | [-0.01, 0.01] |  | -0.01** | [-0.02, -0.01] |  | 0.00 | [ 0.00, 0.01] |  | 0.00 | [ 0.00, 0.01] |  | 1.00 | [ 0.99, 1.01] |  | 1.01 | [ 1.00, 1.01] |
| Weekend (0 = no, 1 = yes) | 0.00 | [-0.08, 0.09] |  | 0.02 | [-0.06, 0.10] |  | -0.10* | [-0.17, -0.02] |  | -0.11** | [-0.18, -0.05] |  | 0.91* | [ 0.83, 1.00] |  | 0.90* | [ 0.82, 0.99] |
| Accelerometer wear time |  |  |  |  |  |  |  |  |  |  |  |  | 1.04** | [ 1.01, 1.06] |  | 1.06*** | [ 1.04, 1.08] |
| Provided support agent | 0.06** | [ 0.02, 0.10] |  | 0.01 | [-0.03, 0.05] |  | -0.04* | [-0.08, 0.00] |  | -0.01 | [-0.04, 0.02] |  | 1.04 | [ 1.00, 1.08] |  | 1.08*** | [ 1.04, 1.13] |
| Provided support target | 0.01 | [-0.03, 0.05] |  | 0.05** | [ 0.01, 0.09] |  | -0.01 | [-0.05, 0.02] |  | -0.05** | [-0.08, -0.01] |  | 1.11*** | [ 1.06, 1.16] |  | 1.09*** | [ 1.05, 1.13] |
| Time spent together | 0.00 | [ 0.00, 0.01] |  | 0.00 | [ 0.00, 0.01] |  | -0.01** | [-0.01, 0.00] |  | 0.00 | [ 0.00, 0.01] |  | 1.00 | [ 0.99, 1.00] |  | 0.99** | [ 0.99, 1.00] |
| *Level 2 (between-person)* |  |  |  |  |  |  |  |  |  |  |  |  |  |  |  |  |  |
| Persuasion agent | 0.27 | [-0.29, 0.83] |  | 0.55 | [-0.03, 1.14] |  | -0.05 | [-0.55, 0.44] |  | -0.62* | [-1.13, -0.11] |  | 0.75 | [ 0.49, 1.14] |  | 0.96 | [ 0.63, 1.46] |
| Pressure agent | 0.12 | [-0.80, 1.03] |  | -0.77 | [-1.73, 0.19] |  | 0.31 | [-0.50, 1.12] |  | 0.67 | [-0.16, 1.49] |  | 1.93 | [ 0.93, 4.00] |  | 1.39 | [ 0.69, 2.80] |
| Accelerometer wear time |  |  |  |  |  |  |  |  |  |  |  |  | 0.99 | [ 0.94, 1.05] |  | 1.04 | [ 0.98, 1.10] |

|  | Positive Affect | | | | |  | Negative Affect | | | | |  | MVPA | | | | |
| --- | --- | --- | --- | --- | --- | --- | --- | --- | --- | --- | --- | --- | --- | --- | --- | --- | --- |
|  | Agent*^a^* | |  | Target*^b^* | |  | Agent*^a^* | |  | Target*^b^* | |  | Agent*^c^* | |  | Target*^d^* | |
| **Random Effects*^f^*** | *Estimate* | CI_95_ |  | *Estimate* | CI_95_ |  | *Estimate* | CI_95_ |  | *Estimate* | CI_95_ |  | Estimate*^e^* | CI_95_ |  | Estimate*^e^* | CI_95_ |
| *SD* intercept | 0.64 | [ 0.55, 0.75] |  | 0.69 |  |  | 0.59 | [ 0.50, 0.68] |  | 0.60 |  |  | 0.47 | [ 0.39, 0.55] |  | 0.47 | [ 0.40, 0.56] |
| *SD* persuasion agent | 0.28 | [ 0.15, 0.52] |  | 0.21 |  |  | 0.29 | [ 0.19, 0.46] |  | 0.25 |  |  | 0.27 | [ 0.16, 0.46] |  | 0.24 | [ 0.12, 0.46] |
| *SD* pressure agent | 0.28 | [ 0.09, 0.92] |  | 0.11 |  |  | 0.25 | [ 0.08, 0.75] |  | 0.24 |  |  |  |  |  | 0.04 | [ 0.00, 30.85] |
| Cor (intercept, persuasion) | -0.44 | [-0.82, 0.21] |  | -0.39 |  |  | -0.48 | [-0.77, -0.01] |  | -0.39 |  |  | 0.20 | [-0.36, 0.62] |  | -0.49 | [-0.81, 0.25] |
| Cor (intercept, pressure) | 0.28 | [-0.68, 0.88] |  | -0.57 |  |  | 0.44 | [-0.64, 0.94] |  | -0.18 |  |  |  |  |  | 0.67 | [-0.65, 0.68] |
| Cor (persuasion, pressure) | 0.23 | [-0.84, 0.93] |  |  |  |  | -0.95 | [-1.00, 0.93] |  | -0.81 |  |  |  |  |  | -0.97 | [ 0.09, 0.88] |
| **Additional Parameters** |  |  |  |  |  |  |  |  |  |  |  |  |  |  |  |  |  |
| *SD* residual | 0.61 | [ 0.58, 0.64] |  | 0.57 |  |  | 0.53 | [ 0.51, 0.55] |  | 0.47 |  |  |  |  |  |  |  |
| Dispersion |  |  |  |  |  |  |  |  |  |  |  |  | 14.79 | [13.37, 16.36] |  | 13.09 | [ 9.38, 18.27] |
| Autocorrelation (AR1) | 0.20 | [ 0.13, 0.27] |  |  |  |  |  |  |  |  |  |  |  |  |  | 0.30 |  |

*Note.* MVPA = moderate to vigorous physical activity (minutes). Persuasion and pressure used by the agent were measured on a 4-point Likert scale, the other discrete variables on a 6-point scale. Non-binary predictors were centered within- and between-person. Separate models were estimated per outcome and partner. Yellow cells mark estimates relevant for our hypotheses that are no longer significant, and green cells contain estimates relevant to our hypotheses that are significant only in the sensitivity analysis, and not in the main analyses. A total of *N* = 99 couples were included. Total observations: *^a^* *n* = 1325. *^b^* *n* = 1269. *^c^* *n* = 1172 *^d^* *n* = 1174. *^e^* For models with a logarithmic link function, random effects are reported on the log scale, while coefficients and confidence intervals were exponentiated, yielding incidence rate ratios (IRRs), representing multiplicative changes. *^f^* *SD* represents the standard deviation of the random slopes or intercepts. To achieve convergence, some estimates could not be computed.

**p* < 0.05. ***p* < 0.01. ****p* < 0.001.

# References

1. Küng P. wbCorr: Bivariate within- and between-cluster correlations. Published online 2023. Accessed July 5, 2023. https://github.com/Pascal-Kueng/wbCorr

2. Chen T, Chen S, Honda T, Nofuji Y, Kishimoto H, Narazaki K. Longitudinal changes in moderate to vigorous physical activity in community-dwelling older men and women: A 2-year prospective cohort study in Japan. *J Phys Act Health*. 2023;20(9):886-893. doi:https://doi.org/10.1123/jpah.2022-0411

3. Umberson D. Gender, marital status and the social control of health behavior. *Soc Sci Med*. 1992;34(8):907-917. doi:10.1016/0277-9536(92)90259-S

4. August KJ, Sorkin DH. Marital status and gender differences in managing a chronic illness: The function of health-related social control. *Soc Sci Med*. 2010;71(10):1831-1838. doi:https://doi.org/10.1016/j.socscimed.2010.08.022

5. Seidel AJ, Franks MM, Stephens MAP, Rook KS. Spouse control and type 2 diabetes management: moderating effects of dyadic expectations for spouse involvement. *Fam Relat*. 2012;61(4):698-709. doi:https://doi.org/10.1111/j.1741-3729.2012.00719.x

6. Westmaas JL, Wild TC, Ferrence R. Effects of gender in social control of smoking cessation. *Health Psychol*. 2002;21(4):368-376. doi:10.1037/0278-6133.21.4.368

7. Reese-Weber M. Intimacy, communication, and aggressive behaviors: Variations by phases of romantic relationship development. *Pers Relatsh*. 2015;22(2):204-215. doi:https://doi.org/10.1111/pere.12074

8. Masumoto K, Taishi N, Shiozaki M. Age and gender differences in relationships among emotion regulation, mood, and mental health. *Gerontol Geriatr Med*. 2016;2. doi:https://doi.org/10.1177/2333721416637022

9. Novak SA, Webster GD. Spousal social control during a weight loss attempt: A daily diary study. *Pers Relatsh*. 2011;18(2):224-241. doi:https://doi.org/10.1111/j.1475-6811.2011.01358.x

10. Rhodes RE, Blanchard CM, Benoit C, et al. Physical activity and sedentary behavior across 12 months in cohort samples of couples without children, expecting their first child, and expecting their second child. *J Behav Med*. 2014;37(3):533-542. doi:https://doi.org/10.1007/s10865-013-9508-7

11. Berli C, Scholz U. Long-Term and Transfer Effects of an Action Control Intervention in Overweight Couples: A Randomized Controlled Trial Using Text Messages. *Front Psychol*. 2021;12. doi:10.3389/fpsyg.2021.754488

12. Bolger N, Laurenceau JP. *Intensive Longitudinal Methods: An Introduction to Diary and Experience Sampling Research*. Guilford Press; 2013.

13. To QG, Stanton R, Schoeppe S, Doering T, Vandelanotte C. Differences in physical activity between weekdays and weekend days among U.S. children and adults: Cross-sectional analysis of NHANES 2011–2014 data. *Prev Med Rep*. 2022;28. doi:https://doi.org/10.1016/j.pmedr.2022.101892

14. Berli C, Bolger N, Shrout PE, Stadler G, Scholz U. Interpersonal processes of couples’ daily support for goal pursuit: The example of physical activity. *Pers Soc Psychol Bull*. 2018;44(3):332-344. doi:10.1177/0146167217739264

15. Berli C, Schwaninger P, Scholz U. “We feel good”: Daily support provision, health behavior, and well-being in romantic couples. *Front Psychol*. 2021;11. doi:https://doi.org/10.3389/fpsyg.2020.622492

16. Bolger N, Zuckerman A, Kessler RC. Invisible support and adjustment to stress. *J Pers Soc Psychol*. 2000;79(6):953-961. doi:https://doi.org/10.1037/0022-3514.71.5.1006

17. Choi L, Liu Z, Matthews CE, Buchowski MS. Validation of accelerometer wear and nonwear time classification algorithm. *Med Sci Sports Exerc*. 2011;43(2):357-364. doi:https://doi.org/10.1249/MSS.0b013e3181ed61a3
